# Finger pad tophi in gout: a rare presentation*

**DOI:** 10.1590/abd1806-4841.20164466

**Published:** 2016

**Authors:** Asli Aksu Çerman, Ilknur Kivanç Altunay, Kübra Esen Salman, Deniz Tunçel

**Affiliations:** 1 Şişli Hamidiye Etfal Training and Research Hospital, Dermatology Department - Istanbul, Turkey; 2 Şişli Hamidiye Etfal Training and Research Hospital, Pathology Department – Istanbul, Turkey

To editor,

Gout is a common rheumatological disease caused by a disturbance in uric acid metabolism. Tophi develop during most advanced clinical stage of gout, and usually present as firm pink nodules or fusiform swelling, mainly at periarticular sites. However, unusual skin manifestations caused by intradermal and subcutaneous deposition of tophaceous material at locations other than periarticular regions have been reported.^1-3^

**Figure 1:**
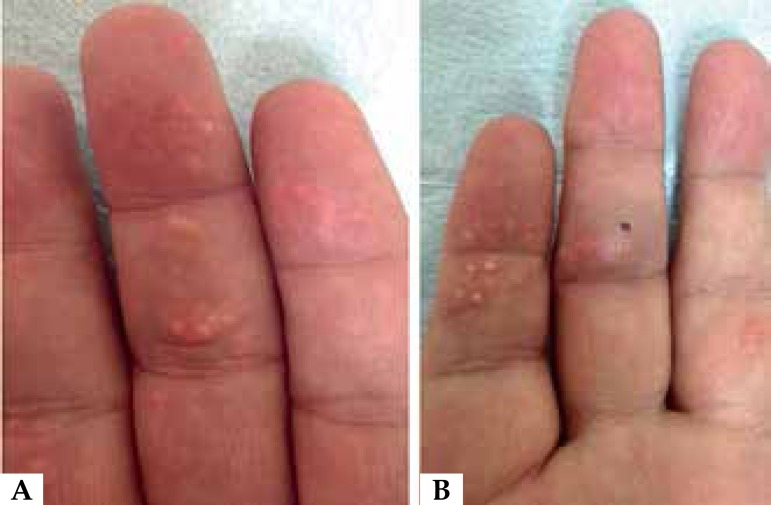
(**A-B**) Multiple, whitish, milia-like, firm papules over the finger pads

**Figure 2:**
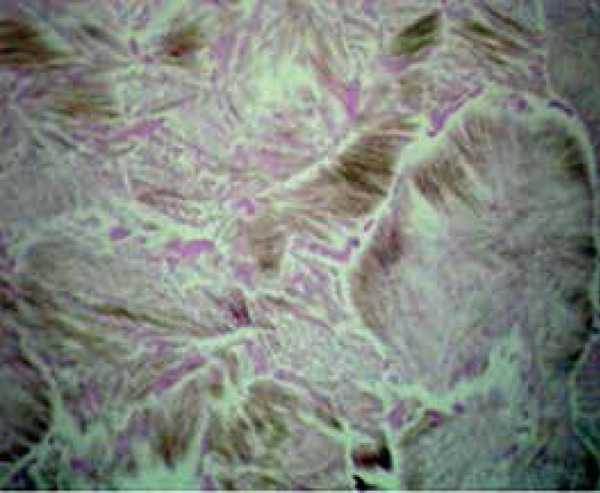
Multiple, needle-like monosodi - um urate crystals were visible under polarized light

We presented a case of a 65-year-old with 2-years history of multiple, whitish, milia-like, firm papules over the finger pads (Figure 1). The patient had intense alcohol use and up to 3 kg/day meat consumption for 20 years. Laboratory tests showed raised levels of uric acid (10.8 mg/dl; normal range 3.4-7 mg/dl), creatinine and cholesterol. Punch biopsy was performed from a typical papule. On histopathological examination, numerous, parallel-lined, needle-like, brown monosodium urate (MSU) crystals and a deposit of pink amorphous material consisting with MSU were seen in the dermis. These crystals showed negatively birefringent under polarized light (Figure 2). Based on the clinical, histopathological and laboratory findings a diagnosis of intradermal tophaceous gout was made. He was referred to rheumatology for management of gout.

The natural history of gout involves four clinical stages; asymptomatic hyperuricemia, acute gouty arthritis, intercritical gout and chronic tophaceous gout. Some atypical forms of tophaceous gout have been described, including bullous, fungating, ulcerative gout, gouty panniculitis and miliarial gout.^1-5^ Intradermal tophi are rare skin manifestations of chronic gout that are characterized by multiple, tiny, superficial, pustule or milia-like, whitish lesions.^3-5^ Rarely they appear at extra-articular sites such as forearms, arms, finger pad, legs, buttock, thigh, penis, vocal cords, epiglottis, tongue and abdominal wall.^1-3^ In our patient, the intradermal gout lesions were restricted to the finger pads. Risk factors predisposing to the development of intradermal gout include renal insufficiency, hypertension, long-term use of furocemid and corticosteroids, long-term duration of disease, obesity, and lack of consistent use of urate-lowering therapy.^1,4^ The present case had some of this risk factors. The differential diagnosis of intradermal tophi includes xanthoma and calcinosis cutis, which can be easily diagnosed by examining fluid in polarized light or performing biopsy.^1,2,5^

Allopurinol and colchicine have been reported to improve intradermal gout, and our patient was referred to a rheumatologist for management but was lost to follow-up.

In conclusion, the incidence of gout is increasing, possibly due to an aging population and eating habits. This case illustrates the importance of considering a rare cutaneous manifestation of gout.
